# Phytochemical and Fungal Bioactive Compounds in the “Brain Health Triad”: A Narrative Review on Neurostimulating, Neurotrophic, and Neuroprotective Synergy

**DOI:** 10.3390/ijms27083607

**Published:** 2026-04-18

**Authors:** Giovanni Luca Cipriano, Ivana Raffaele, Alessia Floramo, Veronica Argento, Maria Francesca Astorino, Maria Lui, Marco Calabrò, Ivan Anchesi

**Affiliations:** 1 IRCCS Centro Neurolesi “Bonino-Pulejo”, Via Provinciale Palermo, Contrada Casazza, 98124 Messina, Italy; giovanniluca.cipriano@irccsme.it (G.L.C.); ivana.raffaele@irccsme.it (I.R.); alessia.floramo@irccsme.it (A.F.); veronica.argento@irccsme.it (V.A.); ivan.anchesi@irccsme.it (I.A.); 2 Department of Biomedical and Dental Sciences and Morpho Functional Imaging, University of Messina, 98125 Messina, Italy; mastorino@unime.it (M.F.A.); mcalabro@unime.it (M.C.)

**Keywords:** *Hericium erinaceus*, *Bacopa monnieri*, L-Theanine, nootropics, neuroprotection, neurorehabilitation, cognitive enhancement, BDNF, neurogenesis, brain health

## Abstract

This narrative review proposes the ‘Brain Health Triad’ as a novel integrative framework for neurorehabilitation and cognitive enhancement, built upon three interdependent biological pillars: neurostimulation, neurotrophy, and neuroprotection. We illustrate how the synergistic interplay between a ‘core triad’ composed of *Hericium erinaceus*, *Bacopa monnieri*, and L-Theanine targets these pillars with high specificity. *Hericium erinaceus* fosters neurotrophy by inducing Nerve Growth Factor (NGF) and Brain-derived neurotrophic factor (BDNF) synthesis through erinacines and hericenones; *Bacopa monnieri* complements this by enhancing neurostimulation and synaptic plasticity via bacosides; and L-Theanine regulates neurotransmitter balance and alpha-wave activity to stabilize the neural signaling environment. This core architecture is further reinforced by adjunctive nootropic clusters—including withanolides, ginkgolides, citicoline, cordycepin, macamides, and fulvic acid—which provide essential support for mitochondrial resilience and the mitigation of amyloid-β and tau toxicities. By synthesizing molecular evidence from the BDNF/TrkB/CREB signaling axis and the Nrf2/NF-κB homeostatic switch, we demonstrate that this multi-target strategy offers a more robust path to neuronal resilience than traditional single-target approaches. We conclude that this integrated model provides a solid framework for future clinical applications in the management of age-related cognitive decline and neurodegenerative diseases.

## 1. Introduction

The global increase in life expectancy has led to a parallel rise in the prevalence of neurodegenerative diseases and age-related cognitive decline [1,2], establishing brain health as a paramount priority in contemporary biomedical research. Despite advancements in understanding neuronal pathophysiology, conventional pharmacological approaches focused on single molecular targets have often demonstrated limited capacity for long-term clinical benefits. This has prompted the scientific community to explore the synergistic potential of pleiotropic natural compounds capable of simultaneously modulating multiple signaling pathways [3,4,5]. Within this context, the present work proposes the ‘Brain Health Triad’ framework, an integrated model synthesizing three fundamental and interdependent biological pillars: neurostimulation, neurotrophy, and neuroprotection [6].

Under this model, neurostimulation functions as the active enhancement of neuronal excitability and synaptic connectivity—processes essential for learning and plasticity [7]. Concurrently, neurotrophy represents the structural maintenance and renewal of the system, where neurotrophic factors such as BDNF and Nerve Growth Factor (NGF) regulate neuronal survival, dendritic morphology, and adult neurogenesis [8,9]. Finally, neuroprotection serves as a critical defense against the destructive cycles of neuroinflammation and oxidative stress, primarily mediated by the activation of molecular hubs such as the nuclear factor erythroid 2-related factor 2 (Nrf2) pathway and the downregulation of nuclear factor-kappa B (NF-κB) [1,10,11].

This review critically examines a strategic selection of phytochemical and fungal bioactive clusters analyzed through the lens of the Triad. Bacosides from *Bacopa monnieri* exhibit significant antioxidant and pro-cognitive properties by modulating mitochondrial function and reducing apoptosis [12,13]. Erinacines and ericerinones isolated from *Hericium erinaceus* are distinguished by their potent inductive activity on NGF and BDNF synthesis, promoting neurite outgrowth [14,15,16,17,18], while L-Theanine adds a layer of anti-stress and neuroprotective effects [19]. Other key compounds include cordycepin from Cordyceps, known for its neuroprotective and anti-inflammatory effects [20,21,22]; ginkgolides from *Ginkgo biloba*, which enhance microcirculation and neuronal resilience [23,24,25]; and citicoline, essential for membrane phospholipid synthesis and neurotransmission support [26,27], while Ashwagandha withanolides mitigate oxidative stress and promote neuronal regeneration [28,29]. The overview is completed by Maca-derived molecules called macamides, providing protection against neurotoxicity [30,31], and fulvic acid from Shilajit, which demonstrates potential in inhibiting tau protein aggregation [32,33]. The integration of these bioactives, alongside their interaction with the gut–brain axis [34,35], aims to demonstrate how a systems biology approach can foster brain resilience superior to traditional reductionist strategies.

## 2. Theoretical Foundations of the Brain Health Triad

**Figure 1:**
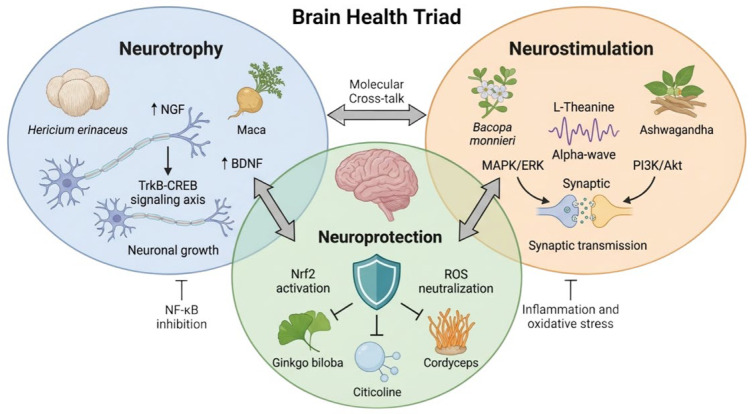
The “Brain Health Triad” conceptual framework. The diagram illustrates the synergistic interplay between neurostimulation, neurotrophy, and neuroprotection, highlighting key bioactive compounds and their primary molecular signaling hubs (e.g., MAPK/ERK, BDNF/TrkB, and Nrf2/NF-κB axes). Bidirectional arrows indicate reciprocal crosstalk between domains; single-headed arrows indicate activation or promotion of molecular pathways or biological effects; blunt-ended lines indicate inhibition or suppression. The central convergence suggests that these domains are functionally interconnected rather than operating as isolated mechanisms.

To understand the synergistic potential of phytochemical and fungal bioactives, one must first establish the molecular architecture of the brain health triad. Each pillar of the triad represents a different temporal and structural aspect of neuronal maintenance (as shown in Figure 1).

### 2.1. Neurostimulation and Molecular Cross-Talk

Neurostimulation refers to the active enhancement of neuronal excitability, synaptic transmission, and the strengthening of neural network connectivity [36]. At its most basic molecular level, neurostimulation involves the activation of signal transduction cascades that translate extracellular stimuli into functional changes in synaptic strength—a process known as synaptic plasticity [7]. The primary drivers of this pillar are the mitogen-activated protein kinase (MAPK)/extracellular signal-regulated kinase (ERK) and the phosphatidylinositol 3-kinase (PI3K)/protein kinase B (Akt) pathways [37,38]. The concept of molecular cross-talk is central to neurostimulation. It describes the interconnected nature of these signaling networks, where the activation of one pathway modulates the sensitivity or activity of another through shared intermediates and feedback loops. For example, the activation of the BDNF-TrkB signaling cascade does not only stimulate ERK phosphorylation but also engages the PI3K-Akt axis, both of which converge on the phosphorylation of the cyclic AMP response element-binding protein (CREB) [8]. CREB serves as a critical transcriptional hub, driving the expression of genes involved in memory consolidation, such as c-fos and various synaptic proteins [8,37].

In this model, each pillar is primarily represented by a specific core bioactive: Neurotrophy is driven by *Hericium erinaceus* through the induction of growth factors; Neurostimulation is facilitated by *Bacopa monnieri* via synaptic plasticity enhancement; and Neuroprotection is ensured by L-Theanine through neurotransmitter modulation and alpha-wave stabilization. This functional architecture is further supported by adjunctive compounds that target mitochondrial health and metabolic resilience.

Recent systems biology research suggests that bioactive compounds do not act as simple agonists but rather as “orchestrators” of this cross-talk, simultaneously modulating multiple upstream regulators to produce a more robust and sustainable cellular response than single-target synthetic drugs [5,39].

### 2.2. Neurotrophy: The NGF and BDNF Signaling Axes

If neurostimulation is the “software” of neuronal activity, neurotrophy is the “hardware” maintenance and upgrade system. Neurotrophic factors (NTFs), particularly NGF and BDNF, are small polypeptide proteins that regulate the survival, growth, and differentiation of neurons [9]. Throughout the adult lifespan, these factors are essential for maintaining the morphological integrity of dendrites and axons, as well as facilitating adult neurogenesis in regions such as the subgranular zone of the hippocampal dentate gyrus [8].

The BDNF-TrkB-CREB pathway represents the central axis of neurotrophy. BDNF binding to its high-affinity receptor, TrkB, triggers receptor dimerization and the autophosphorylation of specific tyrosine residues, which in turn creates docking sites for adapter proteins like Shc and FRS-2 [40]. This initiation activates the downstream PI3K/AKT, MAPK/ERK, and PLCγ cascades. A crucial nuance identified in the recent literature is the role of proBDNF—the precursor form of the protein. While mature BDNF promotes survival and long-term potentiation (LTP) via TrkB, proBDNF binds to the p75 neurotrophin receptor (p75NTR) and can actively promote long-term depression (LTD) or even apoptosis [41]. Therefore, bioactive compounds that either increase BDNF expression or facilitate the conversion of proBDNF to mature BDNF are of significant therapeutic interest [10].

### 2.3. Neuroprotection: Fighting Inflammation and Oxidative Stress

The third pillar, neuroprotection, focuses on shielding the neuronal architecture from the twin threats of chronic neuroinflammation and oxidative stress [4]. These two processes are strongly connected in a self-perpetuating “vicious cycle”: reactive oxygen species (ROS) can activate pro-inflammatory signaling in microglia, while inflammatory cytokines can increase mitochondrial dysfunction and the subsequent generation of more ROS [2]. The primary molecular defense against this cycle is the Nrf2 pathway. Nrf2 is the master regulator of the cellular antioxidant response. Under normal conditions, it is sequestered in the cytoplasm by Kelch-like ECH-associated protein 1 (Keap1), which targets it for degradation [42]. Upon exposure to electrophiles or oxidative stress, Nrf2 dissociates from Keap1, translocates to the nucleus, and binds to antioxidant response elements [21] in the promoter regions of genes such as heme oxygenase-1 (HO-1), superoxide dismutase (SOD), and catalase (CAT) [43,44]. Opposing the Nrf2 pathway is the NF-κB cascade, the primary driver of the pro-inflammatory response. Activation of NF-κB leads to the upregulation of cytokines (Tumor necrosis factor-alpha (TNF-α), IL-1β, IL-6) and enzymes such as inducible nitric oxide synthase (iNOS) and cyclooxygenase-2 (COX-2) [1,5]. The cross-talk between Nrf2 and NF-κB is characterized by reciprocal inhibition; Nrf2 activation can suppress NF-κB by neutralizing the ROS required for its activation and by direct protein–protein interactions that inhibit its transcriptional capacity [45]. Bioactive compounds that can simultaneously upregulate Nrf2 and downregulate NF-κB provide the most comprehensive neuroprotection [43].

## 3. Bioactive Compounds: Core Triad and Adjunctive Clusters

The effectiveness of the brain health triad depends on the specific molecular profiles of the bioactive compounds employed. Each of the clusters reviewed here demonstrates a unique entry point into these pathways, creating opportunities for sophisticated synergy. 

### 3.1. Brain Core Triad Bioactives

The effectiveness of the brain health triad is predicated on a hierarchical selection of bioactive compounds, where a ‘core triad’ serves as the primary driver of the three functional pillars. While numerous phytochemical and fungal clusters exhibit pleiotropic activity, the specific combination of *Hericium erinaceus*, *Bacopa monnieri*, and L-Theanine is distinguished by its capacity to address neurotrophy, neurostimulation, and neuroprotection with high molecular specificity. This core architecture establishes the primary synergistic network across the BDNF/TrkB/CREB and Nrf2/NF-κB signaling hubs, providing a robust foundation for neuronal resilience that is further optimized by the adjunctive clusters discussed later in this review. The following subsections provide a detailed analysis of the molecular profiles and clinical evidence for these three primary bioactives.

#### 3.1.1. Hericenones and Erinacines (*Hericium erinaceus*)

*Hericium erinaceus*, commonly known as Lion’s Mane, is perhaps the most celebrated medicinal mushroom in modern neurobiology due to its ability to induce the synthesis of neurotrophins within the central nervous system [14]. Unlike many synthetic growth factors that cannot cross the blood–brain barrier (BBB), the low-molecular-weight compounds in Lion’s Mane, specifically hericenones from the fruiting body and erinacines from the mycelium, possess excellent BBB permeability [46].

The neurotrophic mechanisms of erinacines, specifically erinacine A, have been investigated through comprehensive preclinical studies, although the exact underlying processes remain to be fully elucidated. Erinacine A stimulates NGF synthesis and induces neuritogenesis via the activation of the TrkA/Erk1/2 signaling pathway in PC12 cells [16]. Interestingly, hericenones and erinacines have been proposed to modulate hypothetically a “Neurotrophic-Epigenetic Axis” by regulating the expression of specific non-coding RNAs (ncRNAs), such as miR-132 and miR-146a. These ncRNAs are known to control the translational efficiency of genes involved in neurogenesis and synaptogenesis, suggesting that Lion’s Mane operates at a deeper level of cellular control than previously thought [39].

Hericenone E isolated from *H. erinaceus* fruiting bodies synergistically potentiates sub-threshold NGF-induced neurite outgrowth in PC12 cells via TrkA-dependent activation of MEK/ERK and PI3K–Akt signaling, thereby functionally amplifying neurotrophin signaling rather than merely increasing NGF levels [47].

Preclinical pharmacokinetic profiling of erinacine A from *H. erinaceus* mycelia demonstrates oral bioavailability and measurable brain exposure in rats, with brain/plasma ratios and half-life compatible with once- or twice-daily dosing after allometric scaling, supporting the translational plausibility of mycelial erinacine-enriched preparations [48].

In neuroprotective models, erinacine A has demonstrated profound efficacy against Parkinson’s disease pathology. In an MPTP-induced rodent model, post-treatment with erinacine A prevented the loss of dopaminergic neurons and rescued motor deficits. This was achieved by activating survival pathways including p21-activated kinase 1 (PAK1), Akt, and MEK, while simultaneously disrupting the IRE1α/TRAF2 cell death cascade that triggers apoptosis [49]. Furthermore, erinacine C has been shown to normalize injury-induced deficits in mild traumatic brain injury (TBI) models through Nrf2-dependent induction of antioxidant enzymes like thioredoxin reductase and superoxide dismutase, while concurrently increasing BDNF expression and CREB phosphorylation [15].

Clinical trials have corroborated these preclinical findings. In elderly patients with mild cognitive impairment (MCI), 16 weeks of Lion’s Mane supplementation significantly improved cognitive scores, with a subsequent decline observed once supplementation ceased, emphasizing its role in maintaining functional neural connectivity [17]. In a longer-term study of mild Alzheimer’s patients, erinacine A-enriched mycelia improved Mini-Mental State Examination (MMSE) scores over 49 weeks, suggesting improved or stabilized cognition and function and preserved white-matter microstructure but longer, larger trials with core AD biomarkers and post-treatment follow-up are needed [50].

An ergothioneine-rich *H. erinaceus* primordium extract prevented age-related recognition memory decline in mice while attenuating hippocampal inflammatory and oxidative markers and upregulating NMDAR1 and mGluR2, indicating preservation of glutamatergic synaptic function alongside modulation of neuroinflammaging [51].

#### 3.1.2. Bacosides (*Bacopa monnieri*)

*Bacopa monnieri*, or Brahmi, is a foundational herb in Ayurvedic medicine with a high density of modern research supporting its use as a cognitive enhancer and neuroprotectant [13,52]. Its primary bioactive compounds, bacosides A and B, are triterpenoid saponins that demonstrate a remarkable ability to modulate neurotransmitter systems and neurotrophic factor expression [53].

The neurostimulating effects of bacosides are largely mediated through the BDNF-TrkB-CREB pathway in the hippocampal dentate gyrus [54,55]. Preclinical studies show that *Bacopa monnieri* extracts (BME) increase markers of cell proliferation like Ki67 and DCX, while significantly enhancing CREB phosphorylation [55]. This leads to increased dendritic branching and the restoration of LTP in models of amnesia and chronic stress.

In the context of type 2 diabetes mellitus research, Bacopa has shown potential in regulating glutamate receptor trafficking. Studies reveal that CDRI-08 can upregulate the expression of the AMPA receptor subunit GluR2 in the hippocampus, effectively normalizing synaptic excitability and preventing excitotoxic injury [12]. In Parkinson’s disease research, Bacopa has demonstrated potential in reducing alpha-synuclein aggregation and protecting dopaminergic neurons by modulating apoptosis (Bax/Bcl-2) [12,52,53]. Furthermore, *Bacopa monnieri* has been shown to reduce tau-mediated cytotoxicity and normalize tau pathology in Alzheimer’s disease (AD) models by inhibiting glycogen synthase kinase-3β (GSK-3β) and restoring Wnt/β-catenin signaling, leading to decreased phospho-tau and total tau levels [56,57]. Clinical data from 22 trials suggests that Bacopa can improve attention, memory retention, and sleep routine while reducing NF-κB phosphorylation and systemic markers of oxidative stress [12].

#### 3.1.3. L-Theanine

L-theanine (N-ethyl-L-glutamine) is a non-protein amino acid predominantly found in the tea plant *Camellia sinensis*, where it can account for more than half of the free amino acid content of the leaf [58]. It is structurally similar to glutamate and glutamine and is well absorbed from the intestine; pharmacokinetic and transport studies show that it crosses the blood–brain barrier via a leucine-preferring amino acid transport system [59].

At the molecular level, L-theanine acts as a structural analog of glutamate, binding to ionotropic glutamate receptors including AMPA, kainate, and NMDA, but with much lower affinity than glutamate itself, and functioning primarily as a glutamatergic modulator rather than a strong antagonist or agonist [19]. It can also interfere with glutamate-related pathways via inhibition of glutamine transporters and modulation of glutamate–glutamine cycling, thereby helping to limit excitatory drive and excitotoxicity in experimental models [60]. In animal and cellular studies, L-theanine administration is associated with increased brain levels of GABA, dopamine, and serotonin, as well as changes in catecholamines and BDNF, consistent with a shift toward a more inhibitory and neuromodulatory tone that underpins its proposed neuroprotective and stress-buffering effects [61].

Within the context of Neurostimulation, L-theanine is distinguished by its capacity to promote a state of “relaxed alertness” rather than sedation. Human EEG and MEG studies show that single oral doses of about 50–250 mg increase alpha-band (8–13 Hz) power, especially over occipital and parietal regions, with effects typically emerging 30–105 min after ingestion [62]. These increases in resting alpha activity are classically linked to a relaxed yet awake state and are often interpreted as “calm attention,” with some studies also reporting improved sustained or selective attention during cognitive tasks, particularly at 200–250 mg doses [63]. However, not all trials find robust cognitive benefits, and alpha enhancement appears more reliable in individuals with higher baseline anxiety or under stress [19].

Clinically, L-theanine supplementation in the range of 200–400 mg/day has shown anti-stress and anxiolytic effects in randomized controlled trials and systematic reviews, including reductions in acute physiological stress markers such as heart rate, sympathetic activation, and salivary cortisol or s-IgA during mental stress tasks, without causing drowsiness [62,63]. Longer-term administration (typically 4–8 weeks at 200–400 mg/day) in stressed or subclinically anxious adults is associated with reduced trait anxiety, improved sleep quality, and some improvements in attention and executive function, though effect sizes are modest and not always superior to placebo [64]. Mechanistically, L-theanine modulates glutamate–GABA balance and increases brain GABA, dopamine, and serotonin, and in animal and translational work can upregulate BDNF expression, supporting a neurotrophic and neuromodulatory profile [65].

By modulating the GABA/glutamate balance and potentially influencing BDNF expression, L-theanine bridges the gap between the neurotrophic induction of *Hericium erinaceus* and the synaptic enhancement of *Bacopa monnieri*, ensuring that the Triad’s pro-cognitive effects are achieved without the autonomic overstimulation often associated with traditional nootropics.

### 3.2. Adjunctive Clusters

#### 3.2.1. Ginkgolides (*Ginkgo biloba*)

*Ginkgo biloba* remains one of the most rigorously studied botanical interventions for cognitive health. Its primary bioactive constituents, the ginkgolides (specifically A, B, C, and J) and bilobalide, are terpene trilactones known for their potent antioxidant and neuroprotective properties [23]. The standardized extract EGb761 has become a clinical benchmark; reviews indicate its efficacy in improving selective attention and memory in healthy and cognitively impaired populations [24], while recent meta-analyses confirm its safety and efficacy specifically in mild dementia [66]. The molecular mechanisms of ginkgolides center on the PI3K/Akt signaling axis and the inhibition of amyloid-β (Aβ) aggregation [67]. Ginkgolide B (GB) has been shown to reduce Aβ production by down-regulating Β-site APP cleaving enzyme 1 (BACE1) expression via AMP-activated protein kinase (AMPK)-dependent signaling in astrocytes [68].

A recent cross-sectional in vitro study highlighted the synergistic potential of ginkgolide B when combined with ferulic acid. This combination was found to restore the expression and functionality of the multifunctional enzyme APE1/Ref-1 across various subcellular compartments, including mitochondria and exosomes. APE1/Ref-1 is essential for the base excision repair (BER) pathway of DNA, and its restoration facilitates the subsequent activation of Nrf2 and CREB, thereby enhancing the cell’s overall capacity for neuroprotection and synaptic plasticity [69]. Clinically, Ginkgo has shown consistent evidence of improving selective attention and long-term memory, although results in healthy subjects under 60 years of age remain more variable [24].

#### 3.2.2. Withanolides (Ashwagandha KSM-66)

*Withania somnifera*, or Ashwagandha, is classified as a premier adaptogen, specifically valued for its ability to modulate the stress response and reduce serum cortisol levels. Its primary bioactives, the withanolides (including withanolide A and withaferin A) and withanosides (such as withanoside IV), provide a multifaceted approach to brain health [28,70,71].

Research has demonstrated that withanolide A can regenerate axons and dendrites and reconstruct pre- and postsynaptic sites in severely damaged cortical neurons [28,70]. Unlike NGF, which primarily induces axonal growth, withanolide A promotes regeneration of both axons and dendrites and the subsequent reconstruction of pre- and postsynaptic sites, as evidenced by restored immunostaining for microtubule-associated protein 2 (MAP2) and the postsynaptic scaffold PSD-95 in amyloid-β–damaged cortical neurons [70].

A standardized sustained-release Ashwagandha root extract (Prolanza™) has been used in clinical research to validate these benefits in stressed adults. Randomized controlled trials confirm that this extract at 300 mg once daily significantly reduces serum cortisol while improving psychological well-being, sleep quality, and cognitive function, particularly memory [72]. Other clinical studies on high-concentration root extracts (such as KSM-66) have confirmed the safety of long-term use (up to 12 months) and improvements in quality of life and organ function safety parameters, although dosages and protocols may vary [73].

At the molecular level, studies indicate that Ashwagandha extracts (such as KSM-66) exert neuroprotective effects by modulating oxidative stress and mitochondrial dysfunction [28]. While one study on in vitro traumatic injury observed neuroprotection via the downregulation of the pro-apoptotic factor Bax rather than direct Nrf2 activation [74], other research in Parkinson’s models demonstrates that the extract significantly increases the activity of antioxidant enzymes like glutathione peroxidase and thioltransferase, and decreases deleterious protein glutathionylation [28]. Furthermore, Withaferin A has been shown to rescue brain network dysfunction and cognitive deficits in Alzheimer’s mouse models [75].

#### 3.2.3. Citicoline (CDP-Choline)

Citicoline (cytidine 5′-diphosphocholine) is a unique endogenous compound that serves as the rate-limiting intermediate in the synthesis of phosphatidylcholine, the most abundant structural phospholipid in neuronal membranes. Upon administration, it dissociates into cytidine and choline, both of which are critical for maintaining the structural integrity and bioenergetics of the brain [76].

The neuroprotective effects of citicoline are rooted in its ability to facilitate membrane repair and inhibit phospholipases that break down cellular barriers during ischemia [77,78]. Recent evidence highlights its role in increasing class III histone deacetylase sirtuin-1 (SIRT1) levels [78]. SIRT1 activation is a major neuroprotective pathway that reduces inflammation and promotes the activity of ADAM metallopeptidase domain 10 (ADAM10) (the α-secretase that processes amyloid precursor protein, APP into non-toxic fragments), thereby reducing the overall burden of Aβ plaques [79].

Clinically, citicoline has demonstrated significant benefit in the recovery of stroke and traumatic brain injury patients. A landmark retrospective cohort analysis assessed in 2025 found that combining citicoline with Cerebrolysin (a neurotrophic peptide complex) led to a trend toward better neurological outcomes in severe TBI patients compared to citicoline monotherapy, although statistical significance was not reached for the primary endpoint. The combination improved patient independence and reduced neurological deficits by simultaneously supporting membrane synthesis and stimulating neuroplasticity [80]. Furthermore, citicoline has been shown to increase the availability of dopamine and norepinephrine, making it a valuable nootropic for improving overall memory performance (especially episodic memory) in both healthy older adults and those with age-associated memory impairment [81].

#### 3.2.4. Cordycepin (Cordyceps)

Cordyceps species, notably Cordyceps militaris, produce cordycepin (3′-deoxyadenosine) as a key bioactive nucleoside derivative. This compound’s structural resemblance to adenosine, lacking only the 3′-hydroxyl group on the ribose moiety, enables it to engage adenosine receptors (A1R and A2AR subtypes of purinergic P1 receptors) in the central nervous system [82,83].

Cordycepin role in the brain health triad is most pronounced in its neuroprotective and pro-energetic effects. It has been shown to alleviate cerebral ischemia-induced memory impairment by inhibiting neuronal apoptosis and suppressing the pathological cell proliferation that often follows brain injury [22]. In models of ischemic stroke, Cordyceps extract enhances BDNF and TrkB receptor expression while significantly reducing infarct size [20,22].

In a global cerebral ischemia model, cordycepin preserved hippocampal CA1 dendritic architecture and long-term potentiation, effects that required adenosine A1 receptor activation and were abrogated by A1R antagonism, indicating that A1R-mediated synaptic modulation is central to its neuroprotective action [83].

In MPTP-induced Parkinsonian mice, cordycepin improved spatial learning and restored hippocampal CA1 LTP while reducing hippocampal adenosine A2A receptor expression and enhancing nigral tyrosine hydroxylase levels, suggesting coordinated dopaminergic and adenosinergic modulation of motor and cognitive circuits [84].

Cordycepin activates AMPK and has been noted to provide neuroprotective effects in Machado–Joseph disease models [20].

Complementarily, in an MPTP Parkinson’s disease model, cordycepin inhibited PI3K/Akt/mTOR and ERK/JNK signaling, promoted autophagy-related protein expression (via ULK1), suppressed microglial M1 polarization and neuronal apoptosis, and attenuated nigrostriatal degeneration [85].

In the context of neuroinflammation, cordycepin is a potent inhibitor of microglial overactivation. It reduces the release of pro-inflammatory cytokines such as TNF-α and Interleukin-1β (IL-1β) by modulating NF-κB signaling [86], as well as Interleukin-6 (IL-6) [85].

#### 3.2.5. Macamides (Maca)

*Lepidium meyenii*, or Maca, is a traditional Andean crop that has gained global attention for its diverse neuroprotective and mood-enhancing effects. Its signature compounds, macamides, are lipid-like molecules derived from N-benzylamides of long-chain fatty acids [30]. The molecular pharmacology of macamides, particularly N-(3-methoxybenzyl)-(9Z,12Z,15Z)-octadecatrienamide (M 18:3), involves activation of the BDNF/TrkB/Akt pathway. In corticosterone-induced neurotoxicity models, M 18:3 ameliorates depressive-like behaviors and hippocampal dendritic structure by elevating p-CREB and BDNF levels [87,88]. Perhaps the most innovative research in this field (2023–2024) involves Maca-derived extracellular vesicles (MACA-EVs). These membrane-enclosed vesicles have been shown to cross the blood–brain barrier and accumulate in the brain, where they promote serotonin (5-HT) synthesis via modulation of the gut–brain axis and subsequently activate the GTP–Cdc42/ERK pathway to support neuronal plasticity [89]. Black maca, the rarest phenotype, has consistently demonstrated superior antioxidant capacity and greater efficacy in improving spatial learning compared to yellow or red varieties [31].

#### 3.2.6. Fulvic Acid

Fulvic acid is a complex mixture of low-molecular-weight organic acids derived from the decomposition of plant matter (humic substances) [33,90]. While often overlooked in classic neuropharmacology, studies highlight its potent ability to inhibit fibril aggregation [33]. Fulvic acid’s primary mechanism of neuroprotection demonstrated in vitro is its ability to directly interfere with the aggregation of tau fibrils and promote their disassembly [33]. Furthermore, recent advancements utilizing nanotechnology have shown promise; fulvic acid-coated iron oxide nanoparticles efficiently inhibit amyloid aggregation in lysozyme models and exhibit no toxicity in SH-SY5Y human neuroblastoma cells, suggesting a novel delivery mechanism for Alzheimer’s therapeutics [91]. Additionally, fulvic acid modulates the immune response by reducing the release of pro-inflammatory cytokines like TNF-α [90]. Future research must bridge the gap between these bench-top molecular inhibitions and clinical neuro-pathological outcomes. To better contextualize these bioactives, their principal molecular targets and functional roles within the brain health triad are summarized in Table 1. Although some mechanistic overlap exists, each cluster retains a relatively distinct profile.

## 4. Synergistic Mechanisms and Molecular Integration

The convergence of these primary phytochemical and fungal clusters onto a select few signaling hubs suggests that the ‘Brain Health Triad’ is not merely a collection of parallel mechanisms but an integrated biological system. The concept of molecular cross-talk provides the framework for understanding how these diverse bioactives work in concert to produce effects greater than the sum of their parts. By targeting overlapping pathways—such as the BDNF-TrkB axis for growth and the Nrf2-ARE pathway for defense—these compounds establish a synergistic network that promotes systemic neuronal resilience rather than isolated biochemical changes

### 4.1. Convergence on the BDNF-TrkB-CREB Signaling Hub

The BDNF/TrkB/CREB pathway serves as the primary integration point for the triad’s neurotrophic and neurostimulating pillars. Within this framework, bacosides and macamides stimulate CREB phosphorylation pathways to drive BDNF transcription [30,57], while erinacines and cordycepin ensure that the TrkB receptor is properly sensitized and expressed to receive these signals [22,93]. This synergy establishes a self-reinforcing feedback loop that begins with initiation, where bacosides reverse amnesia by improving calmodulin activity and attenuating PKA/CREB changes [53], complemented by the capacity of withanolides to rescue brain network dysfunction and cognitive deficits [75].

As the process moves into transcription, phosphorylated CREB (p-CREB) binds to the BDNF promoter—such as promoter IV—thereby increasing the synthesis of BDNF transcripts [94]. The maturity of these factors is then facilitated by erinacines and cordycepin, which maintain neurotrophic levels by increasing both pro-BDNF and BDNF through established mechanisms of synthesis and release [14,95].

To ensure optimal reception of these signals, citicoline supports the synthesis of structural phospholipids like phosphatidylcholine to maintain membrane integrity [27], while ginkgolides activate Akt/Nrf2 pathways to attenuate neuronal injury and support the receptor signaling environment [23]. This leads to amplification, where the resulting BDNF-TrkB binding triggers signaling cascades that drive further CREB phosphorylation, creating a stable, long-term plastic state [96]. This Triad synergy is further exemplified by the crosstalk between the Nrf2 and BDNF pathways. Specifically, the activation of Nrf2-mediated antioxidant defenses by compounds such as erinacines A and C protects glial cells and neurons, creating a self-reinforcing loop where neuroprotection directly fuels neurotrophic support [97].

### 4.2. Nrf2/NF-κB Cross-Talk in Neuroprotection

The reciprocal regulation of the Nrf2 and NF-κB pathways represents a critical node for neuroprotective interventions. Chronic neuroinflammation is often characterized by a state in which NF-κB activity is pathologically elevated while Nrf2 activity is insufficiently low, and Nrf2 deficiency exacerbates microgliosis and astrogliosis [1]. Bioactive compounds capable of acting simultaneously on this switch are essential for breaking the neurodegenerative cycle.

Pachymic acid, a triterpenoid from Poria cocos, protects neuronal cells from hypoxia/reoxygenation injury by down-regulating the pro-inflammatory miR-155 and simultaneously up-regulating Nrf2 and HO-1; Nrf2 knockdown abolishes this cytoprotection, directly linking a fungal metabolite to a miRNA–Nrf2 antioxidant axis in neurons [98]. Erinacine C from *Hericium erinaceus* demonstrates this modulation by activating the Nrf2 pathway. Specifically, erinacine C promotes the binding of Nrf2 to the promoter regions of antioxidant genes such as catalase, thioredoxin reductase, and superoxide dismutase [15].

**Figure 2:**
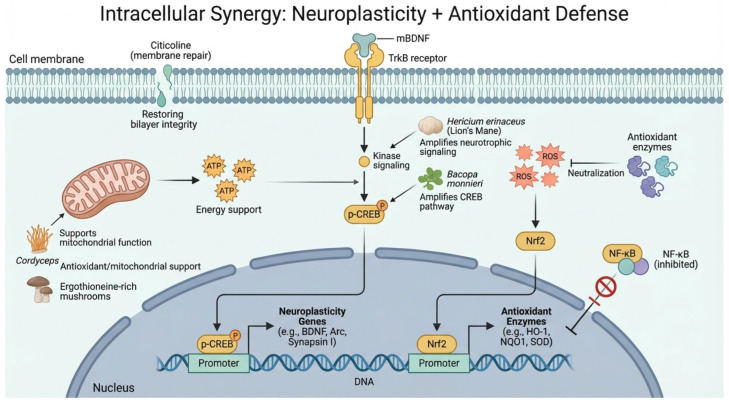
Intracellular synergistic mechanisms of bioactive compounds in neurons. The illustration details membrane restoration (Citicoline), signaling amplification (BDNF/TrkB/CREB axis), mitochondrial bioenergetics (ATP production), and the Nrf2/NF-κB redox-inflammatory switch. Arrows indicate directional signaling or functional support, whereas inhibitory symbols indicate suppression or blockade of a pathway.

Ganoderma triterpenoids (GLTs) and extracts have been shown to reduce oxidative stress and apoptosis in models of amyloid-β toxicity and aging by increasing the production of antioxidant enzymes like SOD and HO-1 via Nrf2 [99,100]. Simultaneously, ginkgolides, such as Ginkgolide B, suppress NF-κB signaling activation, thereby reducing the transcription of pro-inflammatory cytokines [92]. This ‘pincer maneuver’—which enhances internal defenses while reducing inflammatory triggers—provides a more comprehensive shield for the brain. For instance, compounds derived from Inonotus obliquus (such as 3,4-dihydroxybenzalacetone) simultaneously activate the Nrf2 pathway and stabilize mitochondria, exemplifying how a single fungal molecule can co-engage antioxidant and cytoprotective nodes [21] (as shown in Figure 2).

### 4.3. Bioenergetics and Mitochondrial Resilience

A foundational requirement for both neurostimulation and neurotrophy is a reliable supply of cellular energy. Mitochondrial dysfunction, marked by reduced ATP production, loss of membrane potential, and increased ROS leakage, is a common denominator across Alzheimer’s, Parkinson’s, and stroke [101,102]. Several bio-actives focus specifically on this metabolic ground.

Citicoline ensures that mitochondrial membranes have a steady supply of phosphatidylcholine [76], while cordycepin and ginkgolides directly support oxidative phosphorylation and ATP synthesis [103,104]. Recent research has also linked BDNF signaling to mitochondrial health; BDNF has been shown to regulate the respiratory capacity of neurons and promote mitochondrial biogenesis [105].

By inhibiting PI3K/Akt/mTOR and ERK/JNK pathways in MPTP mice, cordycepin enhances autophagy—particularly in microglia—suppressing neuroinflammation and apoptosis. This mechanism preserves nigrostriatal mitochondrial function and dopaminergic neuronal integrity [85].

Ergothioneine, a thiohistidine derivative concentrated in mushrooms, protects human iPSC-derived dopaminergic neurons from oxidative and mitochondrial toxins by reducing mitochondrial ROS, preserving mitochondrial membrane potential, and limiting caspase-dependent apoptosis, thereby maintaining neuronal bioenergetic integrity [106].

By converging on mitochondrial resilience, these compounds ensure that the neuron has the bioenergetic “budget” necessary to build new synapses and repair damaged axons. In addition to signaling modulation, the maintenance of mitochondrial bioenergetics constitutes a third critical axis of this molecular integration. The synergy between clusters like Citicoline and Cordycepin ensures that the neuronal energy demand is met while preserving membrane structural integrity.

## 5. Preclinical Evidence: Insights from Disease Models

This section evaluates the therapeutic efficacy of the ‘Brain Health Triad’ bioactives through a synthesis of specific neuropathological models and clinical outcomes.

### 5.1. Alzheimer’s Disease and Amyloid Toxicity

The pathology of Alzheimer’s disease is characterized by the accumulation of Aβ plaques and tau hyperphosphorylation, both of which lead to severe synaptic loss and neuroinflammation [107]. Recent studies have demonstrated that Ashwagandha-derived withanolides, specifically withaferin A and withanolide A, can significantly reduce Aβ accumulation in transgenic mouse models by facilitating its clearance and protecting neurons from its cytotoxic effects [71,75].

*Bacopa monnieri* has also shown remarkable potential in AD models. In an Alzheimer-like rat model, 4 weeks of Bacopa supplementation improved cognitive behavior and exploratory activity while normalizing tau pathology and decreasing Aβ plaque density. Mechanistically, this was linked to the restoration of Wnt-β-Catenin signaling and the inhibition of Glycogen synthase kinase-3 β (GSK-3β), which effectively “unlocked” the neuronal capacity for synaptic repair [57]. A “crude” polysaccharide extracted from Inonotus obliquus similarly attenuated cognitive deficits and amyloid burden in 3×Tg-AD mice, increasing hippocampal expression of ubiquitin, E1, Parkin, and UCH-L1, and suggesting activation of the ubiquitin–proteasome system as a fungus-mediated mechanism for proteostatic clearance [108]. Similarly, the use of fulvic acid-coated nanoparticles has emerged as a promising strategy for directly disrupting the formation of toxic amyloid fibrils without the systemic side effects associated with synthetic inhibitors [91]. In APP/PS1 models, Ganoderma lucidum triterpenoids improve spatial memory, attenuate hippocampal neuronal apoptosis and tau pathology, and concomitantly enhance the Nrf2–NQO1/HO-1 axis while inhibiting ROCK1/2 signaling, positioning fungal triterpenoids as multi-level modulators of oxidative stress responses and cytoskeletal stability [99]. Furthermore, in APP/PS1 mice, chronic ergothioneine administration improved cognitive performance, reduced Aβ deposition and neuronal damage, attenuated microglial activation and TNF-α levels, and reshaped the gut microbiota alongside short-chain fatty acid profiles and sphingo-/glycerophospholipid metabolism, supporting a gut–brain–lipid axis for this mushroom-derived metabolite [109].

In a D-galactose–induced aging model, a Ganoderma tsugae extract improved locomotor activity and spatial memory, increased brain SOD1, catalase, and BDNF levels, reduced lipid peroxidation, NLRP3 inflammasome activation, and AGE accumulation, and concomitantly preserved hippocampal and cortical dendritic complexity [100].

### 5.2. Parkinson’s Disease and Dopaminergic Integrity

In Parkinson’s disease research, the focus is on preserving the dopaminergic neurons of the substantia nigra. Erinacine A from *Hericium erinaceus* has proven to be a potent protector in MPTP-induced models of PD. It reduces ROS production by approximately 1.3-fold and decreases neuronal apoptosis from 49% to 27%, which in turn rescues motor coordination in vivo [49]. In an MPTP model of Parkinson’s disease, cordycepin reduced motor deficits and spatial memory impairments, restored CA1 hippocampal LTP, increased tyrosine hydroxylase expression in the substantia nigra, and normalized hippocampal adenosine A2A receptor expression, indicating coordinated dopaminergic and adenosinergic modulation of motor and cognitive circuits [84]. Within the same MPTP context, cordycepin also inhibited PI3K/Akt/mTOR and ERK/JNK signaling, promoted the expression of autophagy-related proteins, reduced M1 microglial polarization and nigrostriatal neuronal apoptosis, linking its neuroprotective activity to coordinated remodeling of autophagy and neuroinflammation [85]. Antrodia camphorata polysaccharides provide an additional fungal example, protecting dopaminergic neurons from 6-OHDA toxicity by suppressing ROS production, inhibiting NLRP3 inflammasome activation, and reducing IL-1β release, thereby attenuating motor deficits in murine models of Parkinson’s disease [110]. *Bacopa monnieri* also plays a significant role in PD neuroprotection by reducing oxidative stress. Studies in rats exposed to cigarette smoke (a model of systemic oxidative stress) found that Bacopa treatment significantly increased the brain levels of glutathione, vitamin C, and vitamin E, providing a chemical shield against oxidative brain damage [111]. Furthermore, Ginkgolide A has been shown to enhance the neuroprotective effects of mesenchymal stem cells in 6-OHDA-induced PD models, reducing apoptosis and restoring mitochondrial function [112]. Consistent with these in vivo findings, ergothioneine preserves mitochondrial function and promotes the survival of human iPSC-derived dopaminergic neurons exposed to oxidative and mitochondrial stress, suggesting a translational role for this mushroom-derived metabolite in supporting dopaminergic resilience [106].

### 5.3. Acute Injury and Ischemic Recovery

Traumatic brain injury and stroke involve an acute cascade of excitotoxicity, BBB disruption, and massive ROS generation [39,113]. Citicoline has remained a cornerstone of research in this area. Preclinical models of ischemic stroke demonstrate that citicoline administration reduces infarct volume and improves neurological scores by preventing the breakdown of membrane phospholipids and reducing the buildup of free fatty acids [77]. The combination of citicoline with other bioactives has yielded even more impressive results. In a murine model of ocular hypertension a model for glaucoma-related neuronal injury, the combined administration of citicoline and Coenzyme Q10 (CoQ10) mitigated retinal nerve fiber layer thickening and showed trends toward preserving electroretinography function [114]. In models of TBI, erinacine C has been shown to normalize behavior and reduce brain inflammation by upregulating Nrf2-dependent genes and increasing the expression of antioxidant enzymes and BDNF, facilitating a faster and more complete recovery of neuronal and microglial function [15].

## 6. Clinical Human Evidence and Human Studies

### 6.1. Cognitive Performance in Healthy and Aging Populations

In healthy aging populations, the focus is on maintaining executive function, memory, and attention. *Ginkgo biloba* (EGb761) remains the most clinically validated botanical for this purpose, with consistent evidence supporting its ability to improve long-term verbal and non-verbal memory, although results are most pronounced in individuals over the age of 60 [25]. Observational data indicate that higher habitual mushroom intake is associated with better performance on memory measures and global cognitive tests in older adults, showing a dose–response relationship that remains significant after adjustment for socioeconomic and dietary confounders [115]. Ashwagandha (KSM-66) has emerged as a leader in managing stress-related cognitive decline. A 2025 prospective observational study found that 12 months of KSM-66 administration significantly improved Quality of Life (QoL) and clinical global impression scores in adults, with an excellent safety and tolerability profile [73]. Similarly, a double-blind, placebo-controlled trial of citicoline (500 mg/day for 12 weeks) in healthy older adults with age-associated memory impairment showed significant improvements in episodic memory (Paired Associate test), and overall composite memory scores [81]. In healthy young adults, a standardized *Hericium erinaceus* supplementation produced modest acute effects on aspects of executive function and, after 28 days, reduced perceived stress, suggesting measurable central effects even in the absence of baseline cognitive impairment [116].

### 6.2. Interventions in Mild Cognitive Impairment and Dementia

For populations already experiencing clinical cognitive decline, such as those with MCI or early-stage Alzheimer’s, bioactive compounds offer a potential adjunct to standard therapies. Lion’s Mane mushroom has shown clinically meaningful improvements in cognitive function scores in MCI patients over 12–16 week periods [17]. In more advanced cases, erinacine A-enriched mycelia have demonstrated the ability to sustain these improvements for up to 49 weeks, a significant duration for a natural intervention in a progressive disease [50].

A more recent trial using an erinacine A–enriched mycelial supplement reported improvements in cognitive ability in healthy older adults when adjusting for baseline scores and covariates, strengthening the clinical relevance of *H. erinaceus* cyathane diterpenoids at practical oral dosing [117].

Regarding *Bacopa monnieri*, meta-analyses indicate potential improvements in attention and reaction times, particularly in healthy subjects or those with mild memory complaints [13]. However, a recent systematic review focused specifically on dementia due to Alzheimer’s disease concluded that current evidence for Bacopa’s clinical efficacy in this population remains of very low certainty, despite preclinical data suggesting multi-targeted effects on neuroinflammation and hippocampal protection [118].

In parallel, regarding ergothioneine, studies note that lower plasma levels are observed in patients with MCI and dementia compared to healthy controls, and dietary intake of mushrooms is associated with reduced risk of cognitive impairment [119]. Recent supplementation research investigates mechanisms such as the activation of neurotrophic pathways like the BDNF–TrkB axis, which may underlie cognitive improvements [120].

### 6.3. Emerging Applications: Stroke, TBI, and Mood

The clinical utility of the brain health triad extends to acute neurological events and mood disorders. Citicoline has been extensively tested as an adjunct therapy for ischemic stroke and TBI. A retrospective cohort analysis published in 2025 noted that previous meta-analyses indicated citicoline significantly improved patient independence scores following head injury [80]. Furthermore, this 2025 study found that the combination of citicoline and Cerebrolysin in sTBI patients showed a tendency toward better neurological outcomes, even though the treatment group exhibited more severe baseline injury profiles [80].

In the realm of mood and psychiatric disorders, several bioactives have shown antidepressant-like effects. For instance, Lion’s Mane (*Hericium erinaceus*) has been studied for its ability to alleviate symptoms of depression and anxiety, likely by reversing the reduction in BDNF in the hippocampus—a mechanism associated with neuroplasticity [18,93]. Probiotics and polyphenols that modulate the gut–brain axis have also been shown to influence mood; for example, specific probiotic strains have been reported to reduce depression scores in patients with irritable bowel syndrome (IBS) and modulate brain activation patterns [34], while preclinical models suggest gut microbiota modulation can influence central BDNF levels [34].

### 6.4. Clinical Evidence on L-Theanine: Stress Modulation to Neuropsychiatric Support

Human clinical trials on L-theanine have explored a broad therapeutic range, spanning from healthy adults under acute stress to patients with complex neuropsychiatric conditions such as major depressive disorder, anxiety, obsessive–compulsive disorder (OCD), and schizophrenia [64]. Overall, L-theanine demonstrates a favorable safety profile at dosages typically ranging between 200 and 400 mg/day [121]. Clinical outcomes show modest but consistent benefits, particularly in the areas of stress reduction, sleep quality improvement, and the enhancement of specific cognitive domains [62].

Regarding acute stress and the state of “relaxed alertness,” administration of 200 mg has been demonstrated to increase EEG and MEG alpha power (8–13 Hz). This neurophysiological change correlates with a subjective reduction in stress and a measurable decrease in salivary cortisol levels during cognitive challenges [63]. In young adults, L-theanine, whether administered alone or in combination with arginine, has been shown to blunt salivary alpha-amylase responses to mental stress tests [122].

Furthermore, a pre-operative randomized controlled trial indicated that L-theanine provides effective anxiolysis with minimal sedation, preserving or even improving cognitive performance when compared to standard pharmacological interventions like alprazolam [63].

The impact of L-theanine on sleep quality and anxiety in non-clinical populations has also been well-documented through long-term intake studies. Stressed but otherwise healthy adults receiving 200 mg/day over four weeks showed significant reductions in depression and trait anxiety scores, alongside improved Pittsburgh Sleep Quality Index (PSQI) ratings, specifically regarding sleep latency and sleep disturbance [64]. Similar findings were reported with a 28-day regimen of 400 mg/day, which enhanced cognitive attention and reduced light sleep stages [121]. While various combinations involving L-theanine and other compounds—such as magnesium, B-vitamins, or rhodiola—also report improved sleep parameters, current evidence suggests that the isolated effect of L-theanine remains a primary driver of the observed anxiolytic response [64].

Life-stage specificity represents another critical dimension of L-theanine research, particularly in pediatric and geriatric populations. In boys diagnosed with ADHD, the administration of 400 mg/day for six weeks significantly improved sleep percentage and efficiency as measured by actigraphy, without the occurrence of major adverse events [123]. Conversely, in older adults aged 50–69, single doses have been shown to acutely improve reaction time and working memory accuracy. Sustained intake over 12 weeks in this demographic further supports executive function and focused attention, highlighting its potential for supporting cognitive aging [124].

Finally, L-theanine has shown significant promise as an adjunctive therapy in clinical psychiatry. In patients with Major Depressive Disorder (MDD), an eight-week regimen improved mood, sleep, and cognitive outcomes, while its combination with sertraline led to higher response and remission rates compared to monotherapy [125]. In chronic schizophrenia, adjunctive treatment with 400 mg/day reduced PANSS negative and general psychopathology scores [126]. Improvements have also been noted in obsession subscores for patients with OCD. However, a critical appraisal of the literature reveals specific limitations; for instance, in Generalized Anxiety Disorder (GAD), dosages up to 900 mg/day did not outperform placebo in primary anxiety measures. This suggests that the therapeutic efficacy of L-theanine may be more specific to acute stress responses and specific neuropsychiatric contexts rather than generalized anxiety conditions [126].

To better frame the evidence discussed in the following sections, the main preclinical and clinical findings are summarized in Table 2 according to disease context, compounds used, and principal observed effects.

## 7. Translational Challenges and the Future of Brain Health

Despite the promising evidence, the journey from “farm to pharma” for natural bioactive compounds faces several critical hurdles that must be addressed to ensure their widespread clinical adoption [128].

### 7.1. Standardization and Quality Control

The most significant challenge in botanical and fungal research is the inherent variability of natural products. The chemical composition of a plant or mushroom can vary dramatically based on its ecotype, growing location, soil composition, and post-harvest processing [129]. For example, Maca (*Lepidium meyenii*) phenotypes—black, red, yellow—exhibit distinct biological effects, and recent profiling confirms the presence of specific bioactive compounds like macamides and alkaloids involved in neuroprotection [31,130]. Recent studies on *Hericium* spp. document substantial variability in the content of hericenones, erinacines, hericerins, and polysaccharides as a function of species, cultivation substrate, and developmental stage, underscoring the need for validated chromatographic profiling and content specifications for Lion’s Mane preparations intended for clinical use [97,131]. To overcome this, researchers are utilizing high-performance liquid chromatography (HPLC) and mass spectrometry (MS) to create “chemical fingerprints” for standardization [132]. Similarly, RP-HPLC-DAD analyses of Inonotus obliquus isolates obtained from different host trees reveal marked differences in triterpenoid profiles (such as betulinic acid and botulin) and associated cytotoxicity, indicating that the botanical origin of the substrate substantially shapes the bioactive chemistry of Chaga-derived products [133].

### 7.2. Bioavailability and Blood–Brain Barrier Penetration

The efficacy of a therapeutic agent is fundamentally constrained by its capacity to reach target tissues at a sufficient concentration. While small, lipid-soluble molecules such as erinacines and macamides can naturally cross the BBB, other compounds encounter significant physiological obstacles. Dedicated pharmacokinetic studies indicate that orally administered erinacine A reaches quantifiable concentrations in the rat brain, demonstrating favorable brain-to-plasma ratios and a half-life compatible with once-daily dosing regimens, which supports a direct central mechanism of action [48]. In contrast, the translational potential of certain potent compounds is frequently hindered by pharmacokinetic limitations. For example, larger, highly polar triterpenoid saponins such as parent bacosides have unfavorable physicochemical properties for CNS drugs, leading to poor intestinal permeability and limited BBB penetration [134]. In contrast, their aglycone and aglycone-derived metabolites show improved CNS drug-like profiles and better predicted brain access [134]. To overcome these pharmacokinetic barriers, advanced delivery approaches are being developed, including nanoencapsulation of bacoside A in PLGA nanoparticles to enhance BBB crossing [53], solid lipid nanoparticles to increase systemic exposure and sustain release of bacoside-rich extracts [135], intranasal thermosensitive gels for direct nose-to-brain delivery of bacoside A 4, and specialized matrix systems such as Polar-Nonpolar-Sandwich technology to stabilize bacosides and control their release [53,136]. Regarding fungal triterpenoids, in vivo rat data indicate that Poria cocos–derived pachymic acid exhibits oral bioavailability and a multi-hour half-life (t1/2 ≈ 4.96 h) with a large volume of distribution, suggesting adequate systemic exposure, although its brain penetration remains to be formally established [117]. The future of neurotherapeutics lies in advanced delivery systems designed to overcome these barriers. These innovations include the use of lipid-based or metallic nanoparticles to encapsulate bioactives, thereby protecting them from degradation and facilitating their transport across the BBB [137]. Additionally, plant-derived extracellular vesicles and exosomes, such as Maca-EVs, represent a natural, low-toxicity delivery system capable of carrying multiple neuroactive metabolites directly to brain cells [89]. Intranasal delivery provides another promising route, bypassing the gastrointestinal tract and the BBB by delivering compounds directly through the olfactory and trigeminal nerve pathways to the brain [3]. Finally, unlike high-molecular-weight polysaccharides, ergothioneine acts as a small molecule that is actively transported by the OCTN1 carrier, accumulating in the brain and cerebrospinal fluid, as confirmed by radiolabeled distribution studies showing its uptake within the central nervous system [138].

### 7.3. The Gut–Brain–Microbiome Axis

An emerging frontier in the 2024–2026 research is the role of the gut microbiome in mediating the effects of the brain health triad. Many bioactives act as prebiotics, altering the composition of the gut microbiota to favor beneficial strains like Bifidobacterium and Lactobacillus. These microbes produce short-chain fatty acids (SCFAs) and other metabolites that can cross the BBB and stimulate BDNF expression or reduce neuroinflammation [139].

The (1,3)/(1,6)-β-glucan derived from Lentinula edodes provides a paradigmatic example: in high-fat diet–fed mice, it prevented recognition-memory decline by reshaping the gut microbiota, reducing endotoxemia, preserving colonic barrier integrity, and restoring BDNF levels and synaptic ultrastructure in the prefrontal cortex and hippocampus [35]. For instance, Maca-EVs have been shown to improve depressive-like behavior by modulating gut microbiota composition and increasing the abundance of probiotic taxa that produce neuroactive indoles and 5-HT precursors [89]. In a chronic *Toxoplasma gondii* infection model, the *Lentinula edodes* polysaccharide lentinan prevented cognitive deficits, reduced hippocampal microglial accumulation/activation and pro-inflammatory cytokine expression, and preserved neurite and synapse ultrastructure, with normalization of transcriptomic signatures related to neuroinflammation and synaptic function [140].

In parallel, an ergothioneine-rich *Hericium erinaceus* primordium extract attenuated hippocampal markers of inflammation and oxidative stress, improved recognition memory and frailty index in aged mice, and increased glutamatergic receptor expression, suggesting that mushroom preparations may coordinately modulate the gut–brain axis, neuroinflammaging, and synaptic efficiency [51].

This highlights a “systemic triad,” where the health of the gut and the immune system are inseparable from the health of the brain. A recent synthesis of preclinical evidence on mushroom polysaccharides—including those from Hericium, Ganoderma, and Inonotus—supports the view that these macromolecules act as multi-target modulators of the gut–brain axis by converging effects on the microbiota, oxidative-stress control, mitochondrial function, and neuroinflammation across AD and PD models [141].

### 7.4. From Preclinical Synergy to Clinical Validation: The Methodological Gap

The “Brain Health Triad” framework posits that the coordinated use of *Hericium erinaceus*, *Bacopa monnieri*, and L-theanine yields neurobiological effects that are greater than the sum of their individual actions. Preclinical work and bioinformatic analyses increasingly support this hypothesis, showing convergent modulation of key pathways involved in cognitive resilience, including BDNF/TrkB signaling and Nrf2-driven antioxidant defenses [142,143]. However, a major translational gap remains: clinical research on neuroactive phytochemicals is still dominated by monotherapy or poorly characterized multi-ingredient formulations, with almost no rigorously designed, head-to-head trials comparing single agents to defined combinations [144,145]. To scientifically substantiate synergy claims for multi-component dietary supplements in humans, future trials should prioritize factorial designs, which allow efficient estimation of each component’s effect and their interaction, often with lower sample size and resource requirements than multiple separate trials [146,147,148]. Currently, there is a notable absence of multi-arm randomized controlled trials directly comparing this specific triad with each of its individual components. In the broader drug-combination field, isobolographic analysis and Combination Index (CI) metrics are the standard quantitative tools to classify interactions as synergistic, additive, or antagonistic, but they are applied almost exclusively in preclinical or in vitro settings and rarely translated into formal clinical trial endpoints [149,150]. Without dose–response data suitable for isobologram construction or CI calculation in humans, the proposed synergy of this triad remains a mechanistically plausible hypothesis rather than a clinically demonstrated interaction. Future research should bridge this gap by using factorial or multi-arm designs, preclinically informed dose ratios, and multi-modal biomarkers within a PK/PD modeling framework to test whether this multi-target strategy truly outperforms its components given alone [150,151].

### 7.5. Safety Profiles and Herb-Drug Interactions

Despite the generally high therapeutic index of the phytochemicals and fungal bioactives discussed, rigorous assessment of systemic toxicity and herb–drug interactions (HDIs) remains essential for clinical translation. Ashwagandha (standardized root extracts such as KSM-66) and L-theanine have shown good tolerability in preclinical toxicology and multiple human trials, with no serious adverse effects at commonly used doses and high NOAELs in animal studies [152]. However, reports of hepatotoxicity with some Ashwagandha preparations, dose-related gastrointestinal and sedative effects, and theoretical risks in hyperthyroidism and pregnancy, together with the potential for pharmacodynamic and pharmacokinetic HDIs in polymedicated patients, underscore the need for cautious monitoring, especially in older adults and those on CNS-active or hepatically metabolized drugs [153]. Within multi-ingredient nootropic formulations, complex mixtures and overlapping targets (e.g., CYP enzymes, P-gp, neurotransmitter systems) further complicate prediction of HDIs, reinforcing the importance of standardized products, careful medication review, and post-marketing pharmacovigilance [154,155]. A primary clinical concern with *Ginkgo biloba* is the potential for increased bleeding, especially when used together with anticoagulants such as warfarin or antiplatelet agents such as aspirin. A meta-analysis of 18 randomized trials of standardized Ginkgo extracts found no clinically relevant changes in coagulation parameters or platelet aggregation and no signal for an increased bleeding risk compared with placebo [156]. Likewise, controlled studies of EGb 761 in patients, including those taking aspirin or warfarin, did not detect pharmacodynamic interactions or higher rates of bleeding events [157,158]. However, case reports and pharmacovigilance data describe serious bleeding episodes (including intracranial hemorrhage), and observational work in older adults highlights Ginkgo–aspirin/warfarin combinations as the most commonly flagged herb–drug interaction for bleeding [159,160]. Recent hospital data also show that Ginkgo co-prescribing with antiplatelets and NSAIDs is associated with abnormal coagulation tests and a modestly higher bleeding risk [161]. Taken together, current evidence suggests that standardized Ginkgo extracts do not systematically increase bleeding in clinical trials, but prudence is warranted in patients with additional bleeding risk factors or on multiple antithrombotic drugs. Although standardized extracts like EGb 761 are generally well tolerated, their ginkgolide constituents act as PAF-receptor antagonists and can enhance antiplatelet effects at least in experimental settings [162,163]. Clinical trials, however, show no meaningful amplification of aspirin’s platelet-inhibiting effect or bleeding time with EGb 761 co-administration, and only a modest increase in minor bleeding in some real-world cohorts [164,165]. Consequently, Ginkgo–aspirin combinations warrant thoughtful medication review and monitoring, rather than blanket exclusion, particularly in patients with additional bleeding risks [165,166,167]. Furthermore, the limited and largely preclinical evidence on how *Hericium erinaceus* and *Bacopa monnieri* modulate cytochrome P450 (CYP) enzymes contrasts with the well-documented CYP induction or inhibition seen with several other medicinal herbs, leaving their human interaction profiles insufficiently defined [168,169]. This uncertainty supports proactive pharmacovigilance when these agents are incorporated into multi-target regimens, to mitigate the risk of unforeseen systemic toxicity or altered drug metabolism.

## 8. Conclusions

The “Brain Health Triad” framework presented herein represents a departure from traditional reductionist pharmacology, offering an integrated biological model that addresses the multifaceted nature of neurodegeneration. By synthesizing the pleiotropic effects of eight primary bioactive clusters—bacosides, withanolides, erinacines, ginkgolides, citicoline, cordycepin, macamides, and fulvic acid—this review demonstrates that systemic neuronal resilience is best achieved through the simultaneous modulation of neurostimulation, neurotrophy, and neuroprotection. The molecular convergence of these diverse compounds onto the BDNF-TrkB-CREB signaling hub and the Nrf2/NF-κB homeostatic equilibrium suggests that multi-target synergistic networks are likely superior to single-target interventions in restoring synaptic plasticity and mitigating amyloid-β and tau pathologies. Furthermore, the integration of mitochondrial bioenergetics and the gut–brain–microbiome axis underscores a holistic requirement for brain health, where cellular energy budgets and metabolic stability are inseparable from cognitive function.

Moving forward, the field must navigate critical translational hurdles, particularly regarding the standardization of phytochemical fingerprints and the enhancement of blood–brain barrier penetration through advanced delivery systems. To advance beyond preclinical observations, it is imperative to implement “factorial design” clinical trials that specifically evaluate the co-administration of complementary compounds, such as *Hericium erinaceus* and *Bacopa monnieri*, utilizing multi-modal biomarkers and real-time neuroimaging to validate synergistic outcomes. As global life expectancy increases, the transition toward personalized neuro-phytotherapy offers a sustainable and scientifically rigorous path to optimize cognitive longevity and resilience across the human lifespan.

## Figures and Tables

**Table 1:** Key Bioactive Compound Clusters of the “Brain Health Triad”: Natural Sources, Molecular Targets, and Primary Mechanisms of Action.

Natural Source	Bioactive Compounds	Primary Mechanism of Action and Benefits	Ref.
*Hericium erinaceus* (Lion’s Mane)	Hericenones, Erinacines	Highly permeable to the blood–brain barrier. Actively stimulate NGF and BDNF synthesis, promoting neurite outgrowth and neurogenesis.	[14,16,46,47]
*Bacopa monnieri* (Brahmi)	Bacosides (e.g., A and B)	Modulate the BDNF-TrkB-CREB axis in the hippocampus. Reduce oxidative stress, apoptosis, and prevent tau-mediated cytotoxicity by inhibiting GSK-3β.	[54,55,56,57]
*Camellia sinensis* (Green Tea)	L-Theanine (N-ethyl-L-glutamine)	Promotes alpha-wave activity (8–13 Hz) for relaxed alertness. Modulates the glutamate/GABA balance, reduces acute heart rate/stress responses, and protects against excitotoxicity.	[62,63]
*Withania somnifera* (Ashwagandha)	Withanolides (e.g., Withanolide A)	Promote regeneration of both axons and dendrites, reconstructing pre- and postsynaptic sites. Modulate mitochondrial dysfunction and reduce cortisol levels.	[28,29,70]
*Ginkgo biloba*	Ginkgolides (A, B, C, J), Bilobalide	Enhance microcirculation and inhibit amyloid-β (Aβ) aggregation by down-regulating BACE1 expression. Suppress inflammation by inhibiting NF-κB signaling.	[67,68,92]
Citicoline (CDP-Choline)	Citicoline (precursor)	Essential for phosphatidylcholine synthesis (cell membranes) and mitochondrial bioenergetics. Activates the neuroprotective SIRT1 pathway, reducing Aβ burden.	[76,77,78,79]
Cordyceps	Cordycepin	Engages adenosine receptors. Inhibits neuronal apoptosis, reduces pro-inflammatory cytokines release, and promotes autophagy to preserve mitochondrial function.	[82,83,85,86]
*Lepidium meyenii* (Maca)	Macamides (e.g., M 18:3)	Activate the BDNF/TrkB/Akt pathway, improving dendritic structure. MACA-EVs modulate the gut–brain axis, promoting serotonin synthesis.	[87,88,89]
Shilajit	Fulvic Acid	Directly interferes with the aggregation of tau and amyloid fibrils, promoting their disassembly. Modulates the immune response by reducing pro-inflammatory cytokines	[32,33,90]

Aβ: Amyloid-β; BACE1: β-site APP cleaving enzyme 1; BDNF: Brain-derived neurotrophic factor; CREB: Cyclic AMP response element-binding protein; GABA: Gamma-aminobutyric acid; GSK-3β: Glycogen synthase kinase-3 β; MACA-EVs: Maca-derived extracellular vesicles; NGF: Nerve growth factor; SIRT1: Sirtuin-1; TrkB: Tropomyosin receptor kinase B.

**Table 2:** Synthesis of Preclinical and Clinical Evidence for Bioactive Synergy in Neurodegenerative Diseases, Cognitive Decline, and Acute Brain Injury.

Condition/Disease	Compounds Used	Key Evidence and Observed Effects	Ref.
Alzheimer’s Disease (AD) and Amyloid Toxicity	Ashwagandha, Bacopa, Fulvic Acid	Reduction in Aβ accumulation and clearance facilitation (Ashwagandha). Normalization of tau pathology and improved cognitive behavior (Bacopa). Inhibition of amyloid fibril formation (Fulvic Acid).	[33,57,71,75,91]
Parkinson’s Disease (PD)	Erinacine A, Cordycepin, Bacopa, Ginkgolide A	Protection of dopaminergic neurons, reduction in apoptosis, and rescue of motor deficits (Erinacine A, Cordycepin). Chemical shielding against systemic oxidative stress (Bacopa).	[49,84,85,111]
Ischemic Stroke and Traumatic Brain Injury (TBI)	Citicoline, Erinacine C	Reduction in cerebral infarct volume and improved neurological scores via membrane repair (Citicoline). Normalization of behavior and reduction in brain inflammation via Nrf2 upregulation (Erinacine C).	[15,77,80]
Mild Cognitive Impairment (MCI)	*Hericium erinaceus* (Lion’s Mane)	Clinically significant improvements in cognitive function scores after 12–16 weeks of supplementation. Improvements sustained long-term (up to 49 weeks) with erinacine A-enriched extracts.	[17,50]
Stress, Mood and Sleep Quality	L-Theanine	Increased alpha-wave power (8–13 Hz) and reduced salivary cortisol/alpha-amylase. Improved sleep latency and efficiency (PSQI/actigraphy). Adjunctive efficacy in MDD (remission rates), Schizophrenia (PANSS), and OCD.	[62,121,125,127]
Healthy Aging and Stress	*Ginkgo biloba*, Ashwagandha, Citicoline, L-Theanine	Improvement in long-term verbal and non-verbal memory (Ginkgo). Significant reduction in serum cortisol, improving memory and sleep quality (Ashwagandha). Significant improvements in episodic memory (Citicoline). Acute enhancement of reaction time, attention, and working memory (L-Theanine).	[24,25,64,72,73,81]

AD: Alzheimer’s Disease; Aβ: Amyloid-β; MCI: Mild Cognitive Impairment; PD: Parkinson’s Disease; TBI: Traumatic Brain Injury; Nrf2: Nuclear factor erythroid 2-related factor 2; Hz: Hertz; PSQI: Pittsburgh Sleep Quality Index; MDD: Major Depressive Disorder; PANSS: Positive and Negative Syndrome Scale; OCD: Obsessive–Compulsive Disorder.

## Data Availability

No new data were created or analyzed in this study. Data sharing is not applicable to this article.

## Abbreviations

The following abbreviations are used in this manuscript:

5-HT	Serotonin (5-hydroxytryptamine)
A1R	Adenosine A1 receptor
A2AR	Adenosine A2A receptor
AD	Alzheimer’s Disease
ADAM10	ADAM metallopeptidase domain 10
AKT/PKB	Protein kinase B
AMPK	AMP-activated protein kinase
Aβ	Amyloid-β
BACE1	Β-site APP cleaving enzyme 1
BBB	Blood–brain barrier
BDNF	Brain-derived neurotrophic factor
CAT	Catalase
COX-2	Cyclooxygenase-2
CREB	Cyclic AMP response element-binding protein
ERK/MAPK	Extracellular signal-regulated kinase
GSK-3β	Glycogen synthase kinase-3 β
HO-1	Heme oxygenase-1
IL-1β/IL-6	Interleukin-1β/Interleukin-6
iNOS	Inducible nitric oxide synthase
Keap1	Kelch-like ECH-associated protein 1
LTP/LTD	Long-term potentiation/Long-term depression
MCI	Mild cognitive impairment
NF-κB	Nuclear factor-kappa B
NGF	Nerve growth factor
Nrf2	Nuclear factor erythroid 2-related factor 2
PD	Parkinson’s Disease
PI3K	Phosphatidylinositol 3-kinase
ROS	Reactive oxygen species
SIRT1	Sirtuin-1
SOD	Superoxide dismutase
TBI	Traumatic brain injury
TNF-α	Tumor necrosis factor-alpha
TrkB	Tropomyosin receptor kinase B
